# COVID-19 Vaccination Uptake Among a Nationwide Sample of People Living With HIV During the Early Phase of Vaccine Rollout in China

**DOI:** 10.3389/fmed.2022.822680

**Published:** 2022-06-13

**Authors:** Jianzhou Yang, Maohe Yu, Gengfeng Fu, Guanghua Lan, Linghua Li, Ying Qiao, Jin Zhao, Han-Zhu Qian, Xiangjun Zhang, Xinchao Liu, Xia Jin, Guohong Chen, Yuan Fang, Zixin Wang, Junjie Xu

**Affiliations:** ^1^Department of Preventive Medicine, Changzhi Medical College, Changzhi, China; ^2^Tianjin Center for Disease Control and Prevention, Tianjin, China; ^3^Jiangsu Provincial Center for Disease Control and Prevention, Nanjing, China; ^4^Guangxi Center for Disease Control and Prevention, Nanning, China; ^5^Guangzhou Eighth People's Hospital, Guangzhou Medical University, Guangzhou, China; ^6^The Second Hospital of Huhhot, Huhhot, China; ^7^Shenzhen Center for Disease Control and Prevention, Shenzhen, China; ^8^SJTU-Yale Joint Center for Biostatistics and Data Sciences, Shanghai Jiao Tong University, Shanghai, China; ^9^Department of Public Health, University of Tennessee, Knoxville, TN, United States; ^10^Peking Union Medical College Hospital, Beijing, China; ^11^AIDS Healthcare Foundation (AHF) China, Beijing, China; ^12^Department of Health and Physical Education, The Education University of Hong Kong, Hong Kong, China; ^13^JC School of Public Health and Primary Care, Faculty of Medicine, The Chinese University of Hong Kong, Hong Kong, China; ^14^Clinical Research Academy, Peking University Shenzhen Hospital, Peking University, Shenzhen, China

**Keywords:** people living with HIV, COVID-19 vaccination uptake, associated factors, socioecological model, China

## Abstract

People living with HIV (PLWH), if infected with Coronavirus Disease 2019 (COVID-19), had an increased risk of mortality compared to people without HIV infection. They are considered as a priority group to receive COVID-19 vaccination. This cross-sectional online survey investigated the prevalence of and factors associated with COVID-19 vaccination uptake among 2740 PLWH aged 18–65 years in eight Chinese metropolitan cities between January and February 2021. As validated by requesting participants to send an image of receipt hiding personal identification, 6.2% of PLWH had taken up COVID-19 vaccination. Participants living in cities where individuals could make an appointment to receive COVID-19 vaccination reported significantly higher uptake than those living in cities without such allowance (11.0 vs. 2.9%, *p* < 0.001). Being a member of priority groups to receive vaccination, concerning about the side effects of COVID-19 vaccination and its interaction with HIV treatment, and exposing to information on the Internet/social media supporting PLWH to receive COVID-19 vaccination were significantly associated with COVID-19 vaccination uptake in both groups of participants. Receiving advice from the staff of community-based organizations supporting COVID-19 vaccination was associated with higher uptake among participants living in cities where individuals could make an appointment to receive such vaccination, while a shortage in COVID-19 vaccine supply was associated with a lower uptake among participants living in other cities. Our findings presented a snapshot of COVID-19 vaccination uptake among PLWH in the early phase of vaccine rollout in China. It provided a knowledge basis to formulate interventions promoting COVID-19 vaccination for PLWH.

## Introduction

Coronavirus Disease 2019 (COVID-19) vaccination and other behavioural preventive measures can help to control the ongoing pandemic. Worldwide, COVID-19 vaccination programs started to rollout in December 2020. China initiated the nationwide COVID-19 vaccination program on December 15, 2020 (1, 2). The program was first scaled up in large cities. At the time when this study was conducted (January to February 2021), two COVID-19 vaccination delivery models were implemented simultaneously in China. Individuals could make an appointment to receive COVID-19 vaccination in some cities, whereas vaccination was mainly arranged by the employers, and did not allow individuals to make an appointment in other cities. People had the right to refuse the arrangement. Since the amount of vaccines was inadequate to cover the entire Chinese population during the early phase of rollout, priority was given to several subgroups with increased risks of COVID-19, including people who were working for pandemic control, border control, public transportation, cold-chain, and healthcare workers, as well as those who needed to travel abroad for work or study. Health risk groups (e.g., older adults, people with chronic conditions or immunodeficiency) were not listed as priority groups during the same period, partly due to the lack of sufficient evidence on immunogenicity and safety. Only two types of inactivated COVID-19 vaccines, the Sinovac CoronaVac and Sinopharm, were available during the study period. There were no differences in the availability or indications for these two inactivated vaccines during the study period. By the end of February 2021, 52.5 million doses of COVID-19 vaccines were administered in China (3).

A recent systematic review and meta-analysis suggested that as compared to people without HIV infection, people living with HIV (PLWH) had comparable risk of COVID-19 and risk of developing severe COVID-19 symptoms (4). PLWH, if infected with COVID-19, had an increased risk of mortality compared to those without HIV infection (4). COVID-19 vaccination was effective and safe for PLWH (5). The World Health Organization (WHO), the United States Department of Health and Human Services, the British HIV Association, and health authorities in Australia strongly recommend PLWH to receive COVID-19 vaccination regardless of their CD4+ T-cell counts (6–9). PLWH is one of the priority groups to receive COVID-19 vaccination in various countries (7–9). At the time when this study was conducted, people with immunodeficiency in China needed to seek advice from doctors about whether they should receive COVID-19 vaccination (10). The China national guideline on COVID-19 vaccination was updated 1 month after this study was completed and recommended COVID-19 vaccination to all PLWH (10).

The effectiveness of COVID-19 vaccination program is dependent on both the vaccines' effectiveness and people's willingness to be vaccinated. International health authorities advocate all people without a contraindication should receive COVID-19 vaccination (11). The WHO identified vaccine hesitancy as one major threat to global health (12). A systematic review reported that COVID-19 vaccine hesitancy was a global phenomenon (13, 14). Globally, the number of new COVID-19 cases is rising. Vaccine hesitancy constitutes a threat to tackling the pandemic (15).

Therefore, it is helpful for governments to plan interventions to reduce vaccine hesitancy. In order to promote the COVID-19 vaccination of PLWH, it is necessary to understand their facilitators and barriers to take up COVID-19 vaccination. However, most studies investigating COVID-19 vaccination hesitancy were conducted among the general population and medical professionals (13, 16), and the findings might not be applicable to PLWH. Our literature review identified three studies investigated the willingness to receive COVID-19 vaccination among PLWH in the United States, France and China (17–19). In France and the United States, the proportion of PLWH who were willing to receive COVID-19 vaccination was comparable to those of the general population (France: 72.3% in PLWH vs. 52–76% in the general population; the United States: 68% in PLWH vs. 44–75% in the general population) (17, 18, 20). Our published study showed that 57.2% of unvaccinated PLWH in China were willing to receive COVID-19 vaccination, which was lower than that of the general population (19, 20). Concerns about their health, and the belief that COVID-19 vaccination should be mandatory and important for people with chronic disease were associated with higher willingness to receive COVID-19 vaccination, while previous history of vaccination refusal, mistrust in public health information, and concerns related to side effects were shown to be barriers (17–19). Receiving advice supportive of COVID-19 vaccination for PLWH was associated with higher willingness to receive such vaccination (19). However, there were no studies looking at the actual uptake of COVID-19 vaccination among PLWH. Factors associated with the willingness and actual uptake of COVID-19 vaccination might be different (21). Investigating the willingness and actual uptake of COVID-19 vaccination has different implications. Understanding the willingness and associated factors would inform planning of future health promotion, while looking at the actual uptake provides a snapshot of the implementation of the vaccination program.

This study applied the socio-ecological model as the conceptual framework to explain factors associated with actual uptake of COVID-19 vaccination among PLWH (22). The model considers determinants of a health behavior at the individual, interpersonal and social-structural levels (22). Interventions addressing determinants of a health behavior at multiple levels are more likely to be successful. Previous studies on COVID-19 supported the applicability of the socio-ecological model (23).

This study investigated the prevalence of COVID-19 vaccination uptake among PLWH in China. This study investigated whether factors at different levels (socio-structural, interpersonal, and individual levels) associated with the actual uptake of COVID-19 vaccination were the same in cities with different vaccination delivery models (allowing and not allowing individuals to make an appointment) to receive such vaccine. Our hypotheses were: (1) prevalence of actual uptake of COVID-19 vaccination would be different in cities with different vaccination delivery models, and (2) factors associated with actual uptake would be different under different delivery models.

## Methods

### Study Design and Context

**Figure 1:**
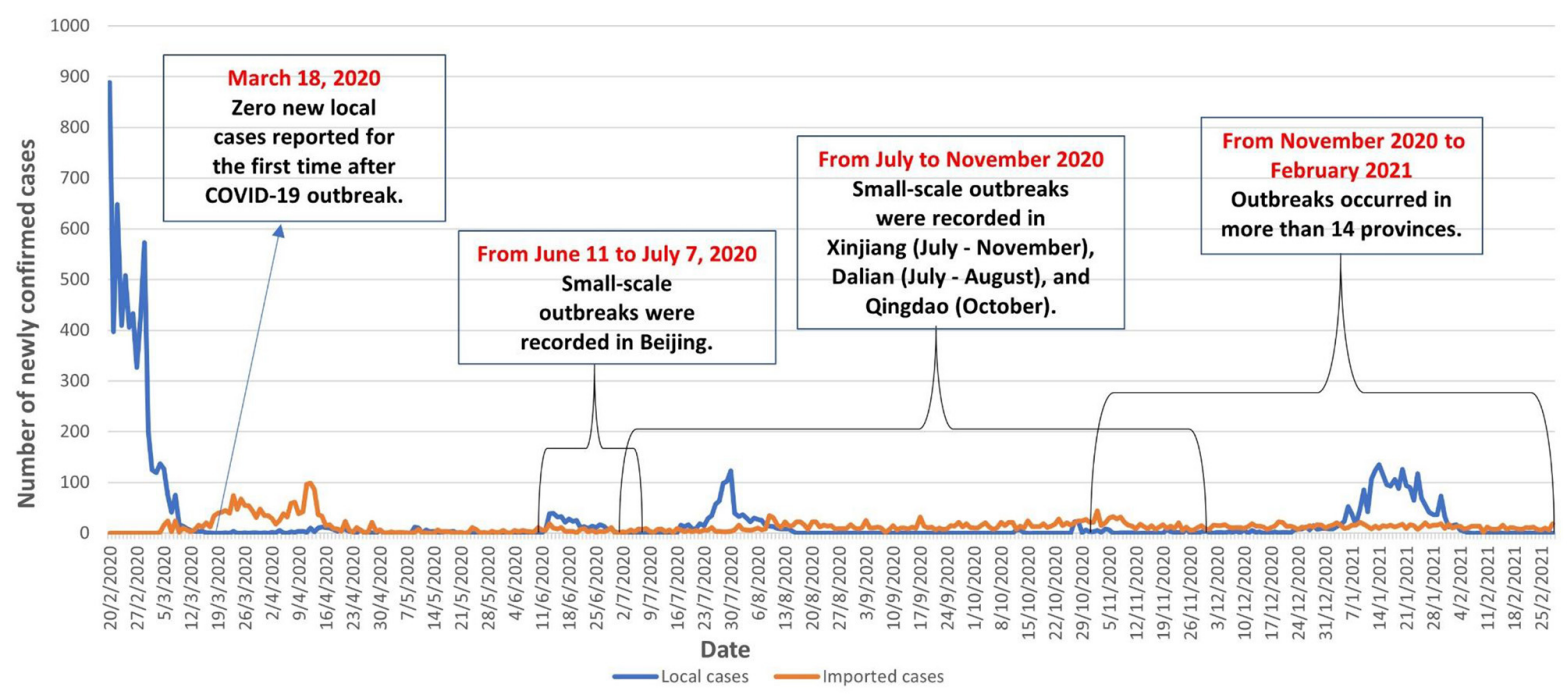
The situation of COVID-19 in China between February 2020 and February 2021.

This is a multicenter cross-sectional online survey conducted between January and February 2021. The study sites covered eight conveniently selected large Chinese cities, including two in the North (Tianjin and Beijing), two in the Northeast (Shenyang, Hohhot), one in the East (Nanjing), and three in the South (Nanning, Guangzhou and Shenzhen). We selected these cities as the COVID-19 vaccination program was first scaled up in these cities. At the time of this study, people in Beijing, Guangzhou, and Shenzhen could make an appointment to receive COVID-19 vaccination. In these cities, people could download a smartphone application developed by the health bureau to schedule their COVID-19 vaccination. In the other five cities, vaccination was arranged by employers and did not allow individuals to make an appointment. Starting from February 2020, the number of daily-confirmed cases continued to decline and the country recorded zero local new cases on March 18, 2020. The number of daily-confirmed local cases remained low (0–12) between March 19 and June 12, 2020. Between June and November 2020, several small-scale outbreaks happened in Beijing (363 cases from June 11 to July 7, 2020), Xinjiang (826 cases between July and September 2020, 78 cases between July and August 2020), Dalian (99 cases between July and August 2020), and Qingdao (14 cases in October 2020). Between November 2020 and February 2021, more than 14 provinces in China reported outbreak and recorded 2286 local cases. The situation of COVID-19 in China between February 2020 and February 2021 was shown in Figure 1.

### Study Population

Study participants were individuals aged 18–65 years who received confirmatory diagnosis with HIV and were living in one of the eight cities at the time of the survey. Individuals who were illiterate and unable to complete the questionnaire survey, unwilling to provide informed consent, or with known contraindications of COVID-19 vaccination (e.g., pregnant and/or lactating women, people with severe allergy to previous vaccination) were excluded. We excluded participants aged over 65 years, as COVID-19 vaccination was not approved for this age group at the time of the survey. The study participants of this study were different from our published study on willingness to receive COVID-19 vaccination (18). This study was conducted among all PLWH, while the published paper focused on a sub-sample of this study who had never received COVID-19 vaccination (18).

### Recruitment and Data Collection

Details of the recruitment and data collection were described by a published study focusing on a subgroup of unvaccinated PLWH of the study participants (19). Eight community-based organizations (CBOs) providing services to PLWH, one in each city, facilitated the recruitment through their networks. These CBOs have been working closely with HIV clinical service providers. WeChat, a live chat application, is the most common communication tool for the CBOs to connect with PLWH clients. CBO staff posted the study information in the WeChat groups involving PLWH clients and invited eligible PLWH to participate. They also sent out reminders through the WeChat groups. The CBO staff and prospective participants were asked not to disseminate the study information to anyone outside the PLWH WeChat groups. Interested PLWH could contact the staff through private WeChat messages or telephone calls. CBO staff screened prospective participants using the eligibility criteria, introduced the study purpose and procedures, answered questions, and explained the confidentiality of study participation. Participation in this study was voluntary, and participants could refuse to answer any of the questions and withdraw from the study at any time without any consequences. Participants signed an electronic consent form sent via WeChat messages. A link to access an online self-administered questionnaire was sent to the consented participants.

The questionnaire was developed using Golden Data, a commonly used, encrypted web-based survey platform in China. Each individual WeChat account was allowed to access the online questionnaire only once to avoid duplicate responses. The Golden Data tool performed completeness check before the questionnaire was submitted. Participants could review and change their responses when they completed the questionnaire. The survey took about 13–15 min to complete. An electronic coupon with a value of 20 Chinese yuan (3.1 US dollar) was sent to the participant upon the completion of a survey. A unique ID was assigned to each participant, which was to delink the study database from personal identifying data. All data collected by the online survey were stored in the Golden Data server and protected by a password. Only the designated research team members had access to the database. Signed electronic consent forms were kept separately from the empirical data and stored in a password-protected computer or a locked cabinet in the same locked office. The Institutional Review Boards of Changzhi Medical College (RT2021003) approved this study.

### Measurements

#### Background Characteristics

Participants were asked to report socio-demographic characteristics, lifestyles (smoking and alcohol drinking), height and weight, presence of chronic conditions, history of other vaccination in the past 3 years, and characteristics related to HIV infection.

#### COVID-19 Vaccination Uptake

Participants reported whether they had taken up any COVID-19 vaccination. Self-reported uptake of COVID-19 vaccination was validated by requesting participants to send the research team an image of their receipt hiding personal identification through WeChat. Some supplementary information was collected from vaccinated participants, including number of doses and types of COVID-19 vaccines received, presence of side effects and severity of such side effects.

#### Socio-Structural-, Individual-, and Interpersonal-level Variables Related to COVID-19 Vaccination

The research team interviewed CDC staff who are responsible for implementing COVID-19 vaccination program about the delivery model and whether there was a shortage in COVID-19 vaccines during the project period in different study sites. Participants were asked whether they belonged to any of the priority groups to receive COVID-19 vaccination listed by the National Health Commission during the project period.

At individual level, this study measured perceptions based on the Theory of Planned Behaviour (TPB), which postulates that in order to perform a behaviour, one would evaluate the pros and cons of the behaviour (positive and negative attitudes), consider whether their significant others would support such behaviour (perceived subjective norm), and appraise how much control one has over the behaviour (perceived behavioural control) (24). The TPB was commonly used to explain a health behaviour and guide the behavioural intervention (25, 26), it has been successfully used to explain the willingness to receive COVID-19 vaccination among Chinese people (27). Four scales were constructed based on the TPB for this study. They were: (1) the five-item Positive Attitude Scale, (2) the five-item Negative Attitude Scale, (3) the four-item Perceived Subjective Norm Scale, and (4) the five-item Perceived Behavioral Control Scale. These scales were formed by summing up individual item score (1 = strongly disagree, 2 = disagree, 3 = neutral, 4 = agree, & 5 = strongly agree).

For interpersonal-level variables, participants were asked whether they received advice given by clinical doctors, CBO staff, friends and family members, and other PLWH regarding COVID-19 vaccination. Participants were also asked the overall opinion regarding COVID-19 vaccination they found on the Internet or social media (responses categories: 1=against taking up COVID-19 vaccination, 2 = no advice/neutral, and 3 = supportive to take up COVID-19 vaccination).

### Sample Size Planning

The target sample size was 2500. We estimated that 10% of the participants received COVID-19 vaccination. Given a statistical power of 0.80 and an alpha value of 0.05 and assuming the uptake rate of COVID-19 vaccination in the reference group (without a facilitating condition) to be 2–8%, the sample size could detect the smallest odds ratios (OR) of 1.47 between people with and without such facilitating conditions (PASS 11.0, NCSS LLS).

### Data Analysis

Self-reported uptake of any COVID-19 vaccination was used as the dependent variable. Since the associated factors under different COVID-19 vaccination delivery models would be largely different, this study investigated factors associated with the dependent variable in two sub-samples. Total scores of the Positive Attitude Scale, the Negative Attitude Scale, the Perceived Subjective Norm Scale, and the Perceived Behavioral Control Scale were used as independent variables in data analysis. Univariate logistic regression models first assessed the significance of the association between each variable and the dependent variable. Variables with *P* < 0.05 in univariate analysis were entered in a multivariate logistic regression model. Crude odds ratios (OR), adjusted odds ratios (AOR) and their 95% confidence interval (CI) are obtained. SPSS version 26.0 (IBM Corp, Armonk, NY) was used for data analysis, with *P*<0.05 considered statistically significant.

## Results

### Background Characteristics

Out of 10,845 PLWH being approached, 8,692 accessed the online survey, and 2740 completed the online survey. Over half of the participants were younger than 40 years (74.4%), were male at birth (94.5%), were currently single (67.8%), received tertiary education (62.9%), were employed full-time (70.5%), had a monthly income <5000 Chinese Yuan (US$ 770) (57.8%), and with basic health insurance only (75.2%). Among the participants, 27.5% were current smokers, 19.6% were current drinkers, 33.3% reported having at least one chronic condition, 6% were using chronic diseases medications, and 23.0% had a history of other vaccination in the past year. Based on self-reported data on height and weight, over one quarter of the participants were overweight (BMI: 24.0–27.9, 22.0%) or obesity (BMI≥28, 5.0%), while 9.0% were underweight (BMI<18.5). Regarding characteristics related to HIV infection, 17.1% of the participants received their diagnosis within – year, 97.4% were on ART, 68.7% reported undetectable HIV viral load, and 47.0% reported their CD4+ T-cell count level was above 500/μl (Table 1).

**Table 1:** Background characteristics of the participants (*n* = 2740).

	***N***	**%**
Age groups (years)		
18–29	838	30.6
30–39	1201	43.8
40–49	513	18.7
50 or above	188	6.9
Gender at birth		
Male	2588	94.5
Female	152	5.5
Gender identity		
Male	2244	81.9
Female	249	9.1
Transgender	239	8.7
Others	8	0.3
Relationship status		
Currently single	1859	67.8
Cohabited/married with a same-sex partner	403	14.7
Cohabited/married with an opposite-sex partner	478	17.4
Highest education level attained		
Junior high or below	441	16.1
Senior high or equivalent	597	21.8
College and above	1702	62.1
Employment status		
Full-time	1931	70.5
Part-time/unemployed/retired/students/others	809	29.5
Monthly personal income, China Yuan (US dollar)		
No fixed income	311	11.4
Below 1,000 (154)	142	5.2
1,000 to 2,999 (154–462)	349	12.7
3,000 to 4,999 (462–770)	783	28.6
5,000 to 6,999 (770–1,078)	528	19.3
7,000 to 9,999 (1,078–1,540)	299	10.9
At least 10,000 (1,540)	328	12.0
Types of health insurance		
No	316	11.5
Basic health insurance only	2060	75.2
Commercial health insurance only	70	2.6
Both basic and commercial health insurance	279	10.2
Others	15	0.5
Study sites		
Beijing	597	21.8
Shenyang	348	12.7
Tianjin	330	12.0
Nanjing	318	11.6
Hohhot	324	11.8
Nanning	305	11.1
Shenzhen	216	7.9
Guangzhou	302	11.0
Current smokers		
No	1986	72.5
Yes	754	27.5
Current drinkers		
No	2204	80.4
Yes	536	19.6
Self-reported BMI		
<18.5	246	9.0
18.5–23.9	1752	63.9
24.0–27.9	604	22.0
≥28	137	5.0
Presence of chronic conditions		
No	1828	66.7
Yes	912	33.3
Chronic diseases medication use		
No	2576	94.0
Yes	164	6.0
History of other vaccination in the past 3 years		
No	2111	77.0
Yes	629	23.0
Time since HIV diagnosis (years)		
≤ 1	468	17.1
2–5	1266	46.2
>5	1006	36.7
On antiretroviral therapy (ART)		
No	72	2.6
Yes	2668	97.4
HIV viral load in the most recent episode of testing		
Undetectable (<50 copies/ml)	1882	68.7
50–200 copies/ml	166	6.1
201–400 copies/ml	71	2.6
>400 copies/ml	144	5.3
Not sure	477	17.4
CD4+ T-cell count in the most recent episode of testing		
>500/μl	1289	47.0
350–499/μl	558	20.4
200–349/μl	271	9.9
<200/μl	89	3.2
Not sure	533	19.5
Self-reported severity of AIDS-related symptoms		
Without any AIDS-related symptoms	1396	50.9
Very mild/mild	896	32.7
Moderate	324	11.8
Severe/very severe	124	4.5

### COVID-19 Vaccination Uptake

Among the participants, 6.2% (*n* = 170) self-reported had taken up at least one dose of COVID-19 vaccine (one dose: *n* = 74, 2.7%; both doses: *n* = 9 6, 3.5%). All these participants were able to provide the receipt for verification. The prevalence of COVID-19 vaccination uptake ranged from 1.4% in Shenyang to 17.3% in Beijing. Among vaccinated participants (n = 170), 62 (36.5%) received Sinopharm, 58 (34.1%) received Sinovac CoronaVac, and 40 (23.5%) of them were not sure about which vaccine they received. Side effects of COVID-19 vaccination were reported by 55.9% of the participants. Common side effects included pain at injection site (*n* = 65, 38.2%), fatigue, headache, dizziness or drowsiness (*n* = 28, 16.5%), muscle pain or joint pain (*n* = 15, 8.8%), and redness, swelling, itching, induration or rash at injection site (*n* = 7, 4.1%). Very few vaccinated participants perceived their side effects to be serious (*n* = 2, 1.2%) (Table 2).

**Table 2:** Supplemental information related to COVID-19 vaccination uptake.

	**N**	**%**
Uptake of at least one dose of COVID-19 vaccination in different study site		
Beijing (*n* = 597)	103	17.3
Shenyang (*n* = 348)	5	1.4
Tianjin (*n* = 330)	11	3.3
Nanjing (*n* = 318)	5	1.6
Hohhot (*n* = 324)	11	3.4
Nanning (*n* = 305)	15	4.9
Shenzhen (*n* = 216)	7	3.2
Guangzhou (*n* = 302)	13	4.3
All participants (*n* = 2740)	170	6.2
Number of doses received (among vaccinated participants, *n* = 170)		
1	74	43.5
2	96	56.5
Side effects of COVID-19 vaccination		
Muscle pain or joint pain	15	8.8
Fatigue, headache, dizziness or drowsiness	28	16.5
Pain at injection site	65	38.2
Redness, swelling, itching, induration or rash at injection site	7	4.1
Itching in non-vaccinated area	2	1.2
Fever (mild, transient)	10	5.9
Nausea, vomiting or diarrhea	1	0.6
Others	6	3.5
Self-reported having serious side effects of COVID-19 vaccination		
No	168	98.8
Yes	2	1.2

### Socio-structural Level, Individual-level, and Interpersonal-level Variables Related to COVID-19 Vaccination

According to CDC staff who were responsible for implementing COVID-19 vaccination program in different study sites, people in Beijing, Guangzhou and Shenzhen could make an appointment to receive COVID-19 vaccination during the project period, while COVID-19 vaccination in the other five cities was arranged by the employers and did not allow individuals to make an appointment. A shortage of COVID-19 vaccines was encountered in Shenyang, Guangzhou, and Shenzhen. Among the participants, 20% identified themselves as priority groups to receive COVID-19 vaccination. The Cronbach's alpha of the scales based on the TPB ranged from 0.83 to 0.92, single factors were identified by exploratory factor analysis, explaining for 61.1–76.4% of total variance (Table 3).

**Table 3:** Responses to survey items measuring socio-structural level, individual-level, and interpersonal-level variables (*n* = 2740).

**Variables**	***N***	**%**
**Socio-structural-level variables**		
Individuals could make an appointment to receive COVID-19 vaccination during the study period		
No	1625	59.3
Yes	1115	40.7
There was a shortage of COVID-19 vaccine in the city where the participants is living during the study period		
No	1874	68.4
Yes	866	31.6
Whether participants belonged to priority groups to receive COVID-19 vaccination in their cities during the study period		
No	2192	80.0
Yes	548	20.0
**Individual-level variables**		
*Perceptions related to COVID-19 vaccination*		
Positive attitudes toward COVID-19 vaccination (% agree/strongly agree)		
COVID-19 vaccination is effective in improving your immune function against COVID-19	1156	42.2
COVID-19 vaccination is effective in reducing your risk of COVID-19	1851	67.6
COVID-19 vaccination is effective in reducing mortality caused by COVID-19	1619	59.1
COVID-19 vaccination is effective in reducing severity of the disease	1667	60.8
Taking up COVID-19 vaccination can make you feel relieved	1713	62.5
*Positive Attitude Scale ^a^, mean (SD)*	*18.4*	*(4.8)*
Negative attitudes toward COVID-19 vaccination (% agree/strongly agree)		
COVID-19 vaccination has severe side effects	1149	41.9
COVID-19 vaccination uptake has significant negative influence on effectiveness of ART	1588	58.0
You have concerns about the risk of exposing your PLWH identity when taking up COVID-19 vaccination	1864	68.0
HIV infection has significant negative influence on effectiveness of COVID-19 vaccination	1552	56.6
The side effects of COVID-19 vaccination are more severer for PLWH than those without HIV infection	1604	58.5
*Negative Attitude Scale ^b^, mean (SD)*	*18.3*	*(5.4)*
Subjective norms related to COVID-19 vaccination (% agree/strongly agree)		
Your family members will support you to take up COVID-19 vaccination	1127	41.1
Your HIV-infected friends will support you to take up COVID-19 vaccination	705	25.7
Medical professionals will support you to take up COVID-19 vaccination	959	35.0
CBO staff will support you to take up COVID-19 vaccination	939	34.3
*Perceived Subjective Norm Scale ^c^, mean (SD)*	*13.3*	*(2.4)*
Perceived behavioral control to take up COVID-19 vaccination (% agree/strongly agree)		
You will take up COVID-19 vaccination even if it will interrupt your daily routine	928	33.9
You will take up COVID-19 vaccination even when you do not feel well	619	22.6
You will take up COVID-19 vaccination even if the side effects would affect your daily activities	681	24.9
You will take up COVID-19 vaccination even if HIV infection would reduce its effectiveness	859	21.3
You will take up COVID-19 vaccination even if it will reduce effectiveness of ART	583	21.3
*Perceived Behavioral Control Scale ^d^, mean (SD)*	*13.1*	*(6.2)*
Interpersonal-level variables		
Advice from doctors regarding COVID-19 vaccination		
Against taking up COVID-19 vaccination	147	5.4
No advice/neutral	2130	77.7
Supportive to take up COVID-19 vaccination	463	16.9
*Mean (SD)*	*2.1*	*(0.5)*
Advice from CBO staff regarding COVID-19 vaccination		
Against taking up COVID-19 vaccination	92	3.4
No advice/neutral	2315	84.5
Supportive to take up COVID-19 vaccination	333	12.2
*Mean (SD)*	*2.1*	*(0.4)*
Advice from friends and family members regarding COVID-19 vaccination		
Against taking up COVID-19 vaccination	40	1.5
No advice/neutral	2644	96.5
Supportive to take up COVID-19 vaccination	56	2.0
*Mean (SD)*	*2.0*	*(0.2)*
Advice from other PLWH regarding COVID-19 vaccination		
Against taking up COVID-19 vaccination	140	5.1
No advice/neutral	2418	88.2
Supportive to take up COVID-19 vaccination	182	6.6
*Mean (SD)*	*2.0*	*(0.3)*
Overall opinion regarding COVID-19 vaccination for PLWH on Internet/social media		
Against taking up COVID-19 vaccination	182	6.6
No advice/neutral	2243	81.9
Supportive to take up COVID-19 vaccination	315	11.5
*Mean (SD)*	*2.0*	*(0.4)*

a *Positive Attitude Scale, five items, Cronbach's alpha: 0.83, one factor was identified by exploratory factor analysis, explaining for 61.1% of total variance;*

b *Negative Attitude Scale, five items, Cronbach's alpha: 0.87, one factor was identified by exploratory factor analysis, explaining for 66.3% of total variance;*

c *Perceived Subjective Norm Scale, four items, Cronbach's alpha: 0.84, one factor was identified by exploratory factor analysis, explaining for 63.4% of total variance;*

d *Perceived Behavioral Control Scale, 5 items, Cronbach's alpha: 0.92, one factor was identified by exploratory factor analysis, explaining for 76.4% of total variance*.

### Factors Associated With COVID-19 Vaccination Uptake

Participants living in cities where individuals could make an appointment to receive COVID-19 vaccination reported significantly higher uptake than those living in cities without such allowance (11.0 vs. 2.9%, *p* < 0.001).

Among participants living in cities where individuals could make an appointment to receive COVID-19 vaccination (*n* = 1115), being a member of priority groups to receive vaccination (AOR: 1.88, 95%CI: 1.15, 3.09), receiving advice from CBO staff supporting COVID-19 vaccination (AOR: 2.06, 95%CI: 1.30, 3.26), and exposing to information on Internet/social media supporting PLWH to receive COVID-19 vaccination (AOR: 2.81, 95%CI: 1.74, 4.52) were associated with higher uptake. Shortage in COVID-19 vaccines (AOR: 0.21, 95%CI: 0.12, 0.38), and having negative attitudes toward COVID-19 vaccination (AOR: 0.85, 95%CI: 0.82, 0.89) were negatively associated with the uptake (Table 4).

**Table 4:** Factors associated with COVID-19 vaccination uptake among participants living in cities/provinces where they can and cannot make an appointment to receive COVID-19 vaccines.

	**Among participants living in cities where individuals could not make an appointment to receive COVID-19 vaccination (*n* = 1625)**		**Among participants living in cities where individuals could make an appointment to receive COVID-19 vaccination (*n* = 1115)**	
	**cOR (95%CI)**	**AOR (95%CI)**	**cOR (95%CI)**	**AOR (95%CI)**
**Background characteristics**				
Age groups (years)
18–29	1.0		1.0	
30–39	1.36 (0.65, 2.84)		1.06 (0.68, 1.65)	
40–49	2.13 (0.95, 4.75)		1.10 (0.64, 1.91)	
50 or above	0.39 (0.05, 3.01)	–	1.06 (0.47, 2.38)	–
Gender at birth				
Male	1.0		1.0	
Female	2.23 (0.97, 5.40)	–	1.37 (0.60, 3.11)	–
Gender identity				
Male	1.0		1.0	
Female	1.74 (0.80, 3.80)		1.48 (0.79, 2.75)	
Transgender	0.27 (0.04, 1.97)		0.81 (0.41, 1.60)	
Others	N.A.	–	N.A.	–
Relationship status				
Currently single	1.0	1.0	1.0	
Cohabited/married with a same-sex partner	2.26 (1.06,4.81)^**^	1.88 (0.85, 4.15)	0.74 (0.42, 1.30)	
Cohabited/married with an opposite-sex partner	2.32 (1.18, 4.56)^**^	2.31 (1.14, 4.70)^*^	1.03 (0.62, 1.71)	–
Highest education level attained				
Junior high or below	1.0		1.0	1.0
Senior high or equivalent	0.34 (0.09, 1.33)		1.47 (0.65, 3.31)	1.47 (0.59, 3.69)
College and above	1.52 (0.67, 3.45)	–	2.55 (1.26, 5.17)^**^	1.76 (0.77, 4.01)
Employment status				
Full-time	1.0	1.0	1.0	1.0
Part-time/unemployed/retired/students/others	0.29 (0.12, 0.69)^**^	0.34 (0.14, 0.82)^*^	0.40 (0.23, 0.69)^***^	0.56 (0.29, 1.10)
Monthly personal income, China Yuan (US dollar)				
No fixed income	1.0		1.0	
Below 1,000 (154)	3.83 (0.70, 21.26)		0.96 (0.19, 4.86)	
1,000 to 2,999 (154–462)	2.04 (0.40, 10.63)		0.96 (0.31, 2.95)	
3,000 to 4,999 (462–770)	3.82 (0.88, 16.61)		1.59 (0.67, 3.75)	
5,000 to 6,999 (770–1,078)	3.00 (0.63, 14.29)		2.31 (0.95, 5.62)	
7,000 to 9,999 (1,078-1,540)	2.91 (0.53, 16.01)		2.31 (0.95, 5.62)	
At least 10,000 (1,540)	5.21 (1.04, 26.24)^*^	–	2.30 (0.97, 5.44)	–
Types of health insurance				
No	1.0		1.0	1.0
Basic health insurance only	3.17 (0.76, 13.21)		2.20 (1.00, 4.86)	1.31 (0.53, 3.20)
Commercial health insurance only	N.A.		0.43 (0.05, 3.56)	0.28 (0.03, 2.80)
Both basic and commercial health insurance	2.10 (0.35, 12.77)		3.29 (1.36, 7.95)^**^	1.85 (0.65, 5.29)
Others	8.55 (0.72, 101.66)	–	N.A.	N.A.
Current smokers				
No	1.0		1.0	
Yes	0.62 (0.30, 1.29)	–	0.83 (0.54, 1.28)	–
Current drinkers				
No	1.0		1.0	
Yes	1.24 (0.62, 2.45)	–	0.98 (0.61, 1.58)	–
Self-reported BMI				
<18.5	1.0		1.0	
18.5–23.9	4.36 (0.59, 32.28)		0.91 (0.45, 1.84)	
24.0–27.9	8.16 (1.08, 61.88)^*^		0.98 (0.46, 2.10)	
≥28	1.85 (0.11, 29.90)	–	1.67 (0.63, 4.43)	–
Presence of chronic conditions				
No	1.0		1.0	
Yes	0.66 (0.34, 1.27)	–	0.90 (0.60, 1.35)	–
Chronic diseases medication use				
No	1.0		1.0	
Yes	0.73 (0.17, 3.04)	–	0.34 (0.11, 1.11)	–
History of other vaccination in the past three years				
No	1.0		1.0	1.0
Yes	1.51 (0.79, 2.90)	–	1.91 (1.29, 2.82)^***^	1.18 (0.74, 1.90)
Time since HIV diagnosis (years)				
≤ 1	1.0		1.0	
2–5	0.72 (0.31,1.68)		1.28 (0.72, 2.28)	
>5	1.26 (0.55, 2.88)	–	1.63 (0.92, 2.88)	–
On antiretroviral therapy (ART)				
No	1.0		1.0	
Yes	1.35 (0.18, 10.00)	—	1.50 (0.35, 6.42)	–
HIV viral load in the most recent episode of testing				
Undetectable (<50 copies/ml)	1.0		1.0	1.0
50–200 copies/ml	1.02 (0.31, 3.37)		0.91 (0.44, 1.89)	1.72 (0.70, 4.30)
201–400 copies/ml	0.80 (0.11, 6.01)		0.21 (0.03, 1.555)	0.49 (0.06, 4.10)
>400 copies/ml	0.95 (0.22, 4.05)		0.44 (0.18, 1.13)	0.66 (0.23, 1.85)
Not sure	0.53 (0.21, 1.36)	–	0.30 (0.14, 0.62)^**^	071 (0.29, 1.76)
CD4+ T cell count in the most recent episode of testing				
>500/μl	1.0		1.0	1.0
350–499/μl	0.29 (0.10, 0.82)^*^		0.66 (0.40, 1.01)	0.99 (0.55, 1.77)
200–349/μl	0.44 (013, 1.46)		0.60 (0.30, 1.20)	0.76 (0.33, 1.77)
<200/μl	N.A.		N.A.	N.A.
Not sure	0.70 (0.33, 1.48)	–	0.37 (0.20, 0.68)^***^	0.62 (0.29, 1.33)
Self-reported severity of AIDS-related symptoms				
Without any AIDS-related symptoms	1.0		1.0	
Very mild/mild	1.13 (0.60, 2.14)		0.95 (0.63, 1.44)	
Moderate	1.04 (0.42, 2.59)		0.70 (0.35, 1.40)	
Severe/very severe	0.52 (0.07, 3.94)	–	0.90 (0.37, 2.18)	–
**Socio-structural-level variables**				
There was a shortage of COVID-19 vaccines in the city where the participants are living during the study period				
No	1.0		1.0	1.0
Yes	0.43 (0.17, 1.09)	–	0.19 (0.12, 0.32)^***^	0.21 (0.12, 0.38)^***^
Whether participants belonged to priority groups to receive COVID-19 vaccination in the city where they are living				
No	1.0	1.0	1.0	1.0
Yes	2.68(1.47,4.89)^**^	2.40(1.26,4.56)^**^	2.18 (1.46, 3.26)^***^	1.88 (1.15, 3.09)^**^
Individual-level variables				
Positive Attitude Scale	1.08 (1.01, 1.15)^*^	1.09(1.01,1.16)^*^	1.04 (1.00, 1.09)^*^	1.05 (0.99, 1.10)
Negative Attitude Scale	0.89 (0.85, 0.93)^***^	0.88 (0.83, 0.93)^**^	0.86 (0.83, 0.89)^***^	0.85 (0.82, 0.89)^***^
Perceived Subjective Norm Scale	1.08 (0.96, 1.21)	–	1.09 (1.01, 1.17)^*^	0.94 (0.84, 1.04)
Perceived Behavioral Control Scale	1.07 (1.03, 1.12)^**^	1.04 (0.99, 1.10)	1.06 (1.03, 1.09)^***^	1.37 (0.86,2.18)
Interpersonal-level variables				
Advice from doctors regarding COVID-19 vaccination	1.72 (0.96, 3.08)	–	1.62 (1.11, 2.36)^**^	1.37 (0.86, 2.18)
Advice from CBO staff regarding COVID-19 vaccination	1.01 (0.50, 2.05)	–	3.55 (2.42, 5.21)^***^	2.06 (1.30, 3.26)^**^
Advice from friends and family members regarding COVID-19 vaccination	1.56 (0.55, 4.43)	–	1.81 (0.94, 3.47)	–
Advice from other PLWH regarding COVID-19 vaccination	1.65 (0.87, 3.12)	–	1.41 (0.92, 2.17)	–
Overall opinion regarding COVID-19 vaccination for PLWH on Internet/social media	2.20 (1.22, 3.99)^**^	2.34 (1.24, 4.41)^**^	3.02 (2.05, 4.44)^***^	2.81 (1.74, 4.52)^***^

* *P < 0.05*,

** *P < 0.01*,

*** *P < 0.001;*

*cOR, crude odds ratios; AOR, adjusted odds ratios obtained from multivariate logistic regression model considering all significant variables in univariate analysis; CI, confidence interval; –, not considered by the model; N.A., not applicable*.

Among those living in cities where individuals could not make an appointment to receive COVID-19 vaccination (*n* = 1625), cohabited/married with an opposite-sex partner (AOR: 2.31, 95%CI: 1.14, 4.70), being a member of priority groups to receive vaccination (AOR: 2.40, 95%CI: 1.26, 4.56), having positive attitudes toward COVID-19 vaccination (AOR: 1.09, 95%CI: 1.01, 1.16), exposing to information on Internet/social media supporting PLWH to receive COVID-19 vaccination (AOR: 2.34, 95%CI: 1.24, 4.41) were associated with higher uptake. Without a full-time job (AOR: 0.34, 95%CI: 0.14, 0.82) and having negative attitudes toward COVID-19 vaccination (AOR: 0.88, 95%CI: 0.83, 0.93) were negatively associated with the dependent variable (Table 4).

## Discussion

To our knowledge, this is one of the first studies investigating COVID-19 vaccination uptake and its associated factors among PLWH in China. As compared to our published study looking at willingness to receive COVID-19 vaccination (18), this study has some different contributions. First, the findings represent the latest estimate of COVID-19 vaccination coverage in this group in the early phase of vaccine rollout, which provided a snapshot of the implementation of COVID-19 vaccination program among PLWH. Second, it addressed the research questions on whether the prevalence of actual uptake and factors associated with actual uptake were the same under different COVID-19 vaccination delivery models. Such information would inform policy makers' decision on which delivery model is more appropriate for PLWH, and provide knowledge basis to develop tailored strategies under each delivery model.

As compared to other countries, the COVID-19 pandemic was stable and under control in China between March 2020 and February 2021. It was possible that Chinese people perceived a lower risk of COVID-19. We found that the COVID-19 vaccination uptake was low (6.2%) and varied across cities (1.4–17.3%). Since there is no data on PLWH in other countries, we were not able to perform between-countries comparison. The COVID-19 vaccination coverage among PLWH was similar to that of the general population in the same cities during the same period. For example, 18% (3.63 million) and 3.6% (0.72 million) of the entire population in Beijing and Shenzhen received COVID-19 vaccination by February 2021, which was similar to our sampled PLWH in these two cities (17.3 and 3.2%) (28). There is a strong need to promote COVID-19 vaccination among both general population and specific sub-population at risk (e.g., PLWH).

Our first hypothesis was supported by the results, as COVID-19 vaccination uptake was much higher under the delivery model which allowed individuals to make an appointment to receive such vaccines. Such model might be easier for PLWH to transform motivation into actual behavior, as they do not have to wait for employer's arrangements. To our knowledge, more cities are switching their delivery model by allowing individuals to make appointments.

Our second hypothesis was partially supported by the results as some factors associated with actual uptake of COVID-19 vaccination was different under different vaccination delivery models. In cities where COVID-19 vaccination was mainly arranged by employers, more attention should be given to people without a full-time job as they could not benefit from such arrangement. Increasing positive attitudes toward COVID-19 vaccination may be an effective strategy to increase vaccination coverage among PLWH in these cities, as it was a facilitator. However, such strategy may not be applicable in cities where individuals could make an appointment to receive COVID-19 vaccination. It is possible that people would consider different factors when they proactively seek a service and when they decide to refuse an arrangement. Shortage in vaccine supply was a barrier of uptake in cities where individuals could make an appointment to receive COVID-19 vaccination, but not for the other delivery model. Allowing individuals to make appointments may result in a surge in the demand. Cities should ensure sufficient vaccine supply before they decide to switch to this delivery model. Shortage of vaccine might overwhelm any other barriers to take up COVID-19 vaccination. The findings also supported that CBO played an important role by motivating PLWH to receive COVID-19 vaccination when individuals could make an appointment to do so. Therefore, it is necessary for the government to empower more capable CBO workers to carry out publicity and education on the necessity and significance of PLWH in the future.

In addition, some similar strategies might be useful for promoting COVID-19 vaccination uptake under both delivery models. At socio-structural level, future intervention should give more attention to PLWH outside the priority groups as they reported lower COVID-19 vaccination uptake. In the initial phase of COVID-19 vaccination rollout and due to the insufficient supply, the government had to give COVID-19 vaccination to individuals at higher risk of COVID-19 first. To address the supply issue, China has rapidly increased its vaccine production capacity (29). It is necessary to shift the focus by emphasizing the importance of herd community targeting the entire population. About half of the participants had negative attitudes related to side effects, risk of exposing their PLWH identity, and potential interactions between COVID-19 vaccination and HIV infection/ART. It is necessary to remove these concerns, as they were barriers of COVID-19 vaccination uptake. Health communication messages should emphasize that ART and COVID-19 vaccination would not have negative impact on each other. Moreover, it is suggested that the health department should establish an online platform for the side effects and antiviral treatment effects of PLWH after receiving the COVID-19 vaccine to eliminate their concerns of COVID-19 vaccination with direct evidence and improve the coverage rate of the vaccine. Positive testimonials from vaccinated PLWH should be useful to remove their concerns related to side effects and privacy. Information about whether PLWH should receive COVID-19 vaccination are mixed on Internet/social media. Similar to the findings in the general population (27), exposure to information on the Internet or social media supporting PLWH to receive COVID-19 vaccination was a facilitator. Previous studies suggested that official web-based media operated by governmental organizations were considered as a credible source of information among Chinese people during the pandemic (30). Government should consider disseminating clear recommendation for PLWH to receive COVID-19 vaccination, and correct the misconception that PLWH could not benefit from such vaccination through these media outlets.

In this study, self-reported COVID-19 vaccination uptake was validated by the research team, which improved the reliability of the primary outcome. The study also has the strengths of covering multiple study sites in different geographic regions of China, a large sample size, and considering variables at all three levels suggested by the socio-ecological model. The findings extended the application of the socio-ecological model and provided empirical insights to inform COVID-19 vaccination promotion strategies. This study also had some limitations. First, this was a cross-sectional survey and could not establish causal relationships. Second, participants were recruited in large Chinese cities. As compared to representative samples of PLWH in China, higher proportion of our participants were male, younger, on ART, and with short duration since HIV diagnosis (31–33). Generalization should be made cautiously to PLWH in China. Third, we were not able to collect information from those who refused to complete the survey. Selection bias existed. Fourth, most items and scales used in this study were self-constructed based on those used in the general population. The reliability of these scales was acceptable. However, they were not used or validated in previous studies. Moreover, participants self-reported whether they belonged to a priority group to receive COVID-19 vaccination. Since we did not ask about participants' occupation and employers, we were not able to confirm whether such self-identification was correct. Furthermore, policies and situation related to COVID-19 vaccination had been changing rapidly; our findings were most applicable to the early phase of COVID-19 vaccination rollout in China. Currently, many low- and middle-income countries with high HIV disease burden were still in the early phase of COVID-19 vaccination rollout (34). Our findings have some reference values for these countries.

## Conclusions

Our findings presented a snapshot of COVID-19 vaccination uptake among PLWH in the early phase of vaccine rollout in China. The socioecological model was useful to explain COVID-19 vaccination uptake among PLWH. The associated factors were not the same under different delivery models. This study provided a knowledge basis to formulate interventions promoting COVID-19 vaccination for PLWH. In order to promote COVID-19 vaccination uptake among PLWH, health authorities should provide clear recommendations of COVID-19 vaccination for PLWH, consider listing PLWH as a priority group to receive COVID-19 vaccination after obtaining sufficient evidence on immunogenicity and safety. Future programs should make use of the settings of hospitals and CBO, as PLWH need to visit these organizations for regular follow-ups. During the follow-up, staff of these organizations could address PLWH's concerns related to the interactions between COVID-19 vaccination and HIV.

## Data Availability Statement

The data presented in this study are available from the corresponding authors upon request. The data are not publicly available as they contain sensitive personal behaviors.

## Ethics Statement

The study protocol was reviewed and approved by the Institutional Review Boards of Changzhi Medical College in Changzhi, China (RT2021003). Participants signed an electronic consent form to participate in the study.

## Author Contributions

ZW, JX, JY, and H-ZQ conceived and designed the research study. JY, MY, GF, GL, LL, YQ, JZ, XZ, XJ, and GC collected the data for the study. ZW and YF analyzed the data and interpreted the results. ZW wrote the first draft of the manuscript. JX, H-ZQ, JY, and XZ revised the manuscript. All authors contributed to the article and approved the submitted version.

## Funding

This study was funded by the Academic technology leader project of Changzhi Medical College (Grant No. XSQ201902) and National Institute of Mental Health of the National Institutes of Health (Award Number R34MH119963). The funders had no role in study design, collection, analysis or interpretation of the data, writing the manuscript, or the decision to submit the paper for publication.

## Conflict of Interest

The authors declare that the research was conducted in the absence of any commercial or financial relationships that could be construed as a potential conflict of interest.

## Publisher's Note

All claims expressed in this article are solely those of the authors and do not necessarily represent those of their affiliated organizations, or those of the publisher, the editors and the reviewers. Any product that may be evaluated in this article, or claim that may be made by its manufacturer, is not guaranteed or endorsed by the publisher.
